# Polychlorinated Biphenyls Water Pollution along the River Nile, Egypt

**DOI:** 10.1155/2015/389213

**Published:** 2015-12-20

**Authors:** Ayman Mohamed Megahed, Hesham Dahshan, Mahdy A. Abd-El-Kader, Amr Mohamed Mohamed Abd-Elall, Mariam Hassan Elbana, Ehab Nabawy, Hend A. Mahmoud

**Affiliations:** ^1^Department of Veterinary Public Health, Faculty of Veterinary Medicine, Zagazig University, Zagazig, Sharkia Governorate 44519, Egypt; ^2^Residue Analysis Department, Central Agricultural Pesticides Lab, Dokki, Giza, Egypt

## Abstract

Ten polychlorinated biphenyl (PCB) congeners were determined in water samples collected along the River Nile using gas chromatography-electron capture detector (GC-ECD). PCB concentrations ranged from 14 to 20 *μ*g/L, which were higher than those reported in previous studies, indicating serious PCB pollution in the River Nile. PCB congener profiles varied depending on the sampling sties. PCB-138 was the predominant congener accounting for more than 18% of total PCBs. The composition of PCB congeners in the water revealed that highly chlorinated PCB technical mixtures such as Aroclor 1254 was the main PCB production historically used in Egypt. An increasing trend in PCB levels from the upper stream to the Nile estuaries was observed. The calculated flux of PCBs indicated that 6.8 tons of PCBs is dumped into the Mediterranean Sea each year from the River Nile. The hazard quotients and carcinogenic risk caused by PCB pollution in the River Nile were above the acceptable level indicating that PCBs in the River Nile water pose adverse health effects for all age groups. Our findings revealed that PCBs possess a serious risk to the Egyptian population that depends mainly on the River Nile as a source of water. Thus, stricter legislation and regulatory controls should be applied to reduce the risk of PCBs in Egypt.

## 1. Introduction

Egypt is an arid country. Millions of Egyptian depend on the River Nile as a source of drinking water and fishing. In recent years, the River Nile is increasingly experiencing pollution from human and industrial wastes. The river supplies 65% of the industrial water needs and receives more than 57% of its effluents [1]. The possible pollutants reaching the river could be persistent organic pollutants (POPs). Polychlorinated biphenyls (PCBs) are one of the 12 groups of POPs originally included in the Stockholm Convention on POPs [2]. PCBs have been used in a wide variety of manufacturing processes in Egypt, especially as plasticizers and insulators, and are widely distributed in the environment [3].

PCB congeners reach water bodies via run-off and/or atmospheric transport. In aquatic ecosystems, PCBs have the potential to bioaccumulate across the food chain, building up in top predators through consumption of contaminated water and biota [4]. Some PCB congeners elicit a divers spectrum of toxic and biochemical responses including body weight loss, immunotoxicity and induction of gene expression [5].

The chemical was banned worldwide in the 1990s because it is highly toxic [6]. In 2007, the Egyptian Environmental Affairs Agency published the framework of the National Implementation Plans (NIPs) about the pollution hazard of POPs in Egypt media. In this context, they concluded that PCBs are still under investigation and information about PCBs in environmental media is limited [7]. Besides, regional monitoring studies of PCBs water pollution are relatively scanty after 2005 as the majority of monitoring reports were published in the interval between 1985 and 2005 [3]. Therefore, our study was constructed to study the occurrence, levels, spatial distribution patterns, and risk assessment of PCB congeners along the River Nile water. Moreover, to supply reference values that could be used for comparison with data from regional, national, or international monitoring programs as the impacts of PCBs on the environment cannot be neglected.

## 2. Materials and Methods

### 2.1. Study Area and Sample Collection

**Figure 1:**
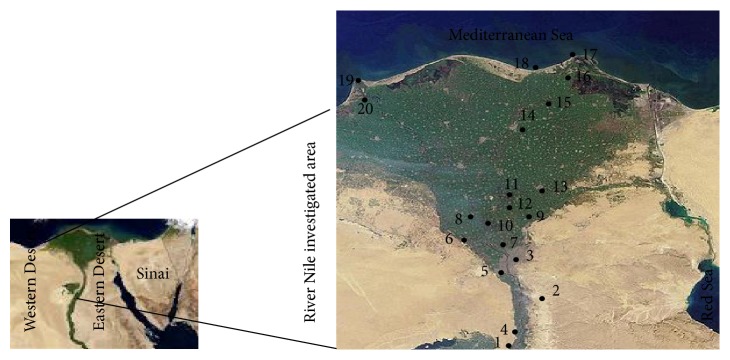
Map of the River Nile showing sites of water sampling: Greater Cairo; 1. Alwasta-Beni Sweif, 2. Helwan, 3. Cairo, 4. Alaeat-Giza, 5. Giza town, 6. Alknater-Giza, 7. Qalubiya and Nile Delta; 8. Monofeya, 9. Belbas, 10. Benha, 11. Alazezea-Menia El Kamh, 12. Menia El Kamh town, 13. Zagazig, 14. Mansoura and Nile estuaries; 15. Fraskour-Damietta, 16. Damietta town, 17. Ras El-bar, 18. Gamasa, 19. Rosetta town, 20. Edfina-Rosetta.

Twenty sampling sites related to three geographical regions along the River Nile were chosen specifically due to its heavy agroindustrial pollution possessed to the river water stream (Figure 1). The first is Greater Cairo, in which the majority of industrial activities are located such as chemical, textile, spinning, steel and galvanizing, food processing, engineering, mining and refraction, and pharmaceuticals industries. The second region is Nile Delta; the majority of agricultural lands are located and the remaining industrial activity rests as vehicles, textiles, fertilizers, food, and detergents. Nile estuaries at Damietta and Rosetta are the third region which is suspected to be heavily polluted by industrial and domestic wastes drained from Greater Cairo and Delta regions. From Greater Cairo and Nile Delta, seven sampling sites were selected for each. Meanwhile, from Nile estuaries, six sites were chosen. Sampling has been carried out during summer, 2013. At each site, three water samples were collected using 2.5 L amber glass bottle at water surface and 50 cm below surface. Before analysis, samples were filtered through 0.45 *μ*m fiber glass filters (Whatman) to remove sand and debris [8].

### 2.2. Chemical Analysis of PCB Congeners

The analytical procedures used in this study are described in detail by Khaled et al. [9]. Briefly, liquid-liquid extraction was used; water sample was extracted twice using 15% methylene chloride in n-hexane. The combined extracts were dried over anhydrous sodium sulfate and concentrated to about 1 mL in a rotary evaporator. For clean-up, the Florisil column was prewashed with 50 mL of n-hexane before the extract was added and this was followed by elution with 60 mL of 30% methylene chloride in n-hexane. The eluate was concentrated using a rotary evaporator until the volume reached 2-3 mL. The residue was dissolved in 2 mL of n-hexane and transferred into autosampler vial for gas chromatography analysis. Analysis was conducted using an Agilent model 6890 gas chromatograph coupled with 63Ni-electron capture detector (GC-ECD) HP-5MS. All the instrumental conditions were reported in Khaled et al. [9]. Chromatographic separation was achieved on a HP-5MS (Agilent, Folsom, CA) capillary column of (30 m × 0.25 mm i.d × 0.25 *μ*m).

### 2.3. Quality Assurance and Quality Control

The individual reference standards used for quantification and identification of PCB congeners were obtained from Dr. Ehrenstorfer GmbH (Augsburg, Germany). All reagents used during the analysis were exposed to same extraction procedures and subsequently run to check for interfering substances. Sample of each series was analyzed in triplicates. The limits of detection (LOD) of PCBs were defined as five times of the signal-to-noise ratio (S/N). The recoveries of PCB congeners ranged between 78 and 98%.

### 2.4. Flux Estimation and Risk Assessment of PCBs

#### 2.4.1. Flux Estimation

According to Zulin et al. [10], the flux of PCB congeners can be estimated using the following equation:

(1) $$\begin{array}{c} F=CQ\times 10^{-12}. \end{array}$$

In this equation, *F* is the flux of congeners from the river to other water bodies (tons per year), *C* is the concentration of congeners recorded in the river water (ng L^−1^), and *Q* is the average annual flow of the river body (m^3^ per year).

#### 2.4.2. Risk Assessment

Risk assessment of the River Nile water was assessed using the hazard quotient and cancer risk assessment. Hazard quotient was calculated according to EPA [11] using the following equations:

(2) $$\begin{array}{c} HQ=\frac{ADD}{RfD}, \end{array}$$

where HQ is the hazard quotient, ADD is the intake exposure level (mg/kg/day), and RfD is the reference doses that have to be consistent (2 × 10^−5^ mg/kg/day). Consider

(3) $$\begin{array}{c} ADD=C\times FI\times \frac{\left(IR\times EF\times ED\right)}{\left(BW\times AT\right)}\;\;\left[\;mg/{kg}^{-1}/d^{-1}\;\right]. \end{array}$$

*C* represents the average concentration of PCBs during the monitoring period (nanogram per cubic decimeter). FI is the fraction ingested (an absolute number in arrange of 0-1). According to previous estimations, FI was assumed to be 0.98. IR represents the daily water intake rates in relation to the age groups according to ECETOC [12] which are as follows: 0–6, 0.3 dm^3^/day; 7–17, 1 dm^3^/day; and adults, 1.4 dm^3^/day. EF is the exposure frequency which is 365 days/year. ED is the exposure duration and it depends on the age group as it is 6 for age group (0–6), 11 (7–17), and 30 adults. BW is the average body weight of 15 kg for the 0–6 age group, 46 kg for the 7–17 age group, and 70 kg for adults [13]. AT is the values of the averaging times. They are expressed in days. For the 0–6 age group AT_0–6_ = 2190, for 7–17 age group AT_7–17_ = 4015, and for adults AT_A_ = 10950 days. Consider

(4) $$\begin{array}{c} \text{Cancer risk}=\frac{C\times DI\times ED}{BW\times AT}\times CSF\times CF. \end{array}$$

According to the risk guidelines of USEPA [14], the cancer risk assessment of PCB congeners via water consumption was calculated. *C* is the concentrations of PCBs in water sample (ng L^−1^); DI is the daily input (L day^−1^): 2 L day^−1^; ED is the exposure duration (year): 30 years; BW is body weight (kg): 60 kg; AT is average life span (year): 70 years; CSF is the cancer slope factor (mg/kg/day)^−1^: 0.07 (mg/kg/day); and CF is the conversion factor: 10^−6^.

### 2.5. Statistical Analysis

Statistical analysis incorporated in the work includes mean of samples and corresponding standard error. Data was subjected to one-way analysis of variance (ANOVA). The calculations were performed using statistical software, SPSS version 17.

## 3. Results and Discussion

### 3.1. PCB Levels in River Water

**Figure 2:**
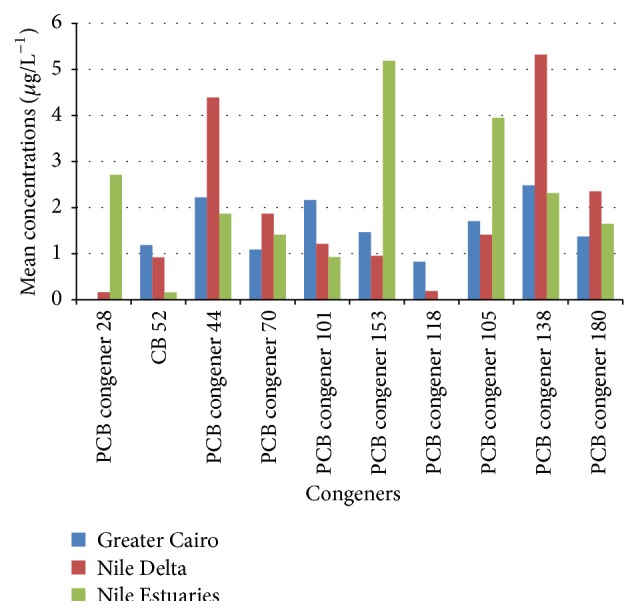
Mean PCB congener concentrations in waters from the different sampling regions.

All investigated PCB congeners were detected and its distribution in water from the three regions sampled is summarized in Table 1. Significant variations in mean concentration were observed between PCB congeners and sampling regions, Figure 2. In water samples that were obtained from Greater Cairo and Nile Delta, PCB congener 138 was predominant with concentration values of 2.482 and 5.322 *μ*g/L^−1^, respectively. However, at Nile estuaries PCB congener 153 showed the highest value of 5.189 *μ*g/L^−1^. The highest concentrations of total 10 PCBs were detected in samples derived from rapid developing cities such as Zagazig city (sampling site S13), Gamasa city (S18), Beni Sweif city (S1), Cairo city (S3), and Ras El-bar city (S17). The main industrial activities there from which hazard effluents are released are textiles, food, detergents, fertilizers, steel, cement, pharmaceuticals, furniture, pesticides, and soap production.

Fluxes in the detected PCB levels in different parts of the River Nile indicate local inputs of contaminants. PCBs pollution is commonly subdivided based on the primary region affected by contamination, creating categories such as River Nile estuaries, Nile Delta, and Greater Cairo water pollution. Fluxes in river water contamination levels are mainly supplied by the estimated 700 industrial facilities along the Nile banks. Highest concentrations of PCBs were measured in Nile water estuaries, where the most industrialized towns in Egypt as Damietta, Rosetta, Ras El-bar, and Gamasa are located.

Based on the contribution of the individual congeners to the total (Σ) PCBs along the River Nile water samples, PCB congener 138 was the highest congener (10.119 *μ*g/L^−1^), followed by PCB congeners 44, 153, 105, 180, 70, 101, 28, and 52. PCB congener 118 was the lowest congener (1.009 *μ*g/L^−1^), Table 1. There was a cumulative effect in contamination levels toward the Nile estuaries as ΣPCBs were increased in upward pattern from Greater Cairo in which ΣPCBs were the lowest (14.503 *μ*g/L^−1^) followed by Nile Delta (18.771 *μ*g/L^−1^). Meanwhile, toward the Nile estuaries ΣPCBs were detected at the highest level (20.166 *μ*g/L^−1^), Table 1 and Figure 4. This could be attributed to POPs including PCBs which are semivolatile compounds, enabling them to move long distances in the atmosphere before deposition occurs. They condense at cooler temperatures, reaching their highest concentrations in the cooler regions: Nile estuaries at Damietta and Rosetta [3].

### 3.2. PCB Congeners Pattern versus Aroclors in Water Samples of the River Nile, Egypt

**Figure 3:**
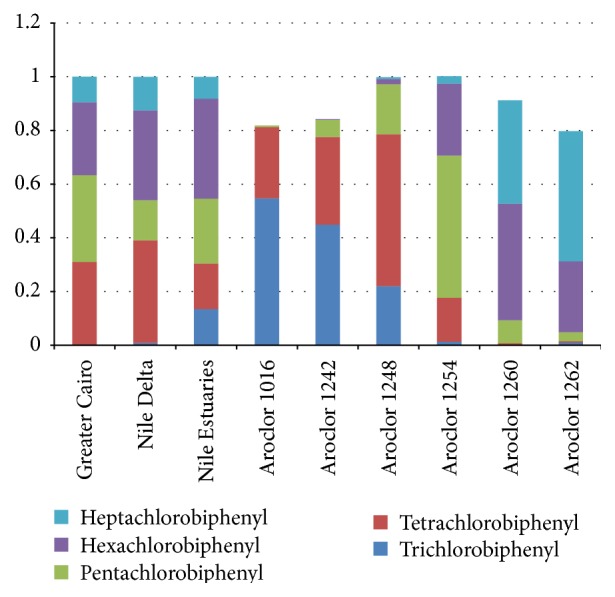
Patterns of PCBs homologues in River Nile water samples, Egypt.

**Figure 4:**
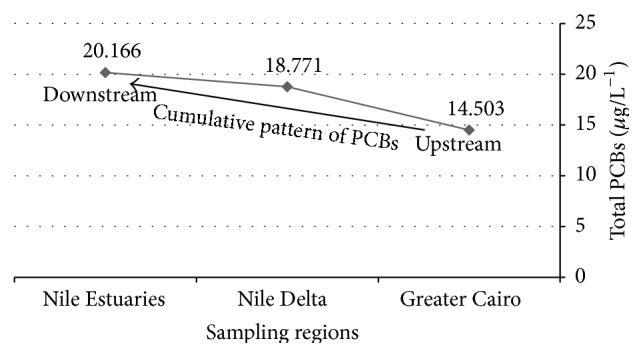
Spatial distribution of total PCBs in waters from the different sampling regions.

The comparison of the patterns of PCBs homologues in water samples with six main commercial PCBs was shown in Figure 3. Along the River Nile sampling regions, PCB congener profiles were dominated by congeners with the tetra-, penta- and hexachlorobiphenyl (CB). Congeners with tri-, tetra-, penta-, hexa-, and hepta-CB were all detected in Nile Delta and Nile estuaries water samples. Meanwhile, tetra-, penta-, hexa-, and hepta-CB were obtained in Greater Cairo samples.

For the lighter Aroclors (1016, 1242, and 1248), tri- and tetra-CB are the most abundant homologue groups. In the heaver ones (1254), penta- and hexa-CB are more abundant, while hepta-CB is the prominent isomer group in Aroclors 1260 and 1262. Based on the previous results and the profiles presented in Figure 3, it can be concluded that the main Aroclor mixtures used in the past in the studied sampling regions should be 1254. Therefore, The PCBs produced in Egypt were mainly composed of highly chlorinated congeners (heavier Aroclors).

### 3.3. Growing Burden of PCBs River Nile Water Pollution

The total level of PCBs in water samples in this study (53.44 *μ*g/L^−1^) was 32-fold higher than the ΣPCBs of similar congeners reported by Wahaab and Badawy [1] in waters samples collected in 1995 from different locations along the River Nile (1637 ng/L^−1^) in Egypt. Furthermore, the PCB concentrations from this study were compared with other studies on river water samples worldwide; in China, Pearl River Estuary deep water column, Middle Reaches of Yangtze River, Tonghui River of Beijing, and Minjiang River Estuary, the total concentration of PCBs were 0.02–14.8 ng L^−1^, 3.77–61.79 ng L^−1^, 31.58–344.9 ng L^−1^, and 204–2473 ng L^−1^, respectively [15]; in USA, Houston Ship Channel, Mississippi River, Delaware River, and raw water of Hudson River, the PCBs level were 0.49–12.5 ng L^−1^, 22.2–163 ng L^−1^, 0.42–1.65 ng/L^−1^, and <9.3–164.3 ng L^−1^, respectively [15]; in surface water bodies in Johannesburg City, South Africa, the PCBs concentrations were ranged from 0.021 to 0.121 ng L^−1^ [16]; and in Ebro River in Spain the concentrations were 43.2–108 ng/L^−1^. Hence, the level of PCBs in the River Nile water, Egypt, was generally at the high end of the global range suggesting that River Nile water may pose a health risk to both human population and animal population.

Moreover, despite the fact that in aquatic ecosystems small amounts of PCBs may be redissolved at the water-sediment interface but mostly tend to partition into sediments and suspended particles [17], we were surprised to found higher PCBs values than that detected in sediment samples in various studies worldwide: the Guanabara Bay in Brazil (range, 18–184 pg g^−1^), Congo River Basin in the Democratic Republic of Congo (<50–1400 pg/g^−1^), Istanbul Strait in Turkey (13–699 pg/g^−1^), Dagu Drainage River in China (9687–22,148 pg/g^−1^), and River Nile, Lake Qarun in Egypt (1480–137,200 pg/g^−1^) [18]. This suggests that an important reason for the high observed values of PCBs in water of the River Nile could be related to the various industrial activities along the banks of the river that dispose hazardous wastes and subsequently runoff of the PCBs into the water body. The major industrial sectors polluting the water of the River Nile are raw and fabricated metals, vehicles, and pharmaceuticals. Other important industries include textiles, pesticides, fertilizers, petrochemicals, cement, paper and pulp, and food processing. About 50% of industrial activity is concentrated in Greater Cairo (Cairo, Giza, Helwan, and Qalubiya) and the remaining rests in the Delta regions and Nile estuaries. Besides, it is estimated that more than 400 factories continue to discharge more than 2.5 million m^3^ per day of untreated effluent into Egypt's river waters [19]. Accordingly, all environmental ecosystems are in hazardous risk as exposure to multitoxicants of different degrees of potency and having either similar or dissimilar mode of action are a toxicological complex issue. Such exposure may adversely affect human health as many POPs including PCBs are believed to be possible carcinogens or mutagens.

### 3.4. Flux Estimation and Risk Assessment

#### 3.4.1. Flux Estimation

In our study, River Nile is feeding the Mediterranean Sea. The fluxes of PCBs from River Nile to the Mediterranean Sea can be calculated using the aforementioned flux estimation equation; total concentration of PCBs at the sites S17, S18, and S19 (before joining with the Mediterranean Sea) was 80760 ng/L^−1^, and annually River Nile flow is 83.660 billion cubic meters. Thus, the annual flux for PCBs from River Nile to the Mediterranean Sea was 6.8 tons per year.

#### 3.4.2. Risk Assessment

There are no criteria for the maximum allowable concentration of PCBs in water in Egypt, but the USEPA [20] set this value for PCBs in drinking water to be 500 ng L^−1^. The total PCBs along the River Nile selected regions were all above the specified values.

Table 2 shows individual average daily doses and hazard quotients due to the contamination of river water with PCBs. According to Table 2 data, the HQs are above unity as all values are dramatically greater than 1. And despite the probable adverse health effects for all age groups, it is unlikely to be proportional to risk. Therefore, cancer risk assessment of PCBs was calculated in the three selected geographical regions (Greater Cairo, Nile Delta, and Nile Estuaries) along the River Nile and valued as 0.014 × 10^−3^, 0.018 × 10^−3^, and 0.02 × 10^−3^, respectively. The accepted level of cancer risk for PCBs water pollution was in 10^−4^–10^−6^ [21]. Hence, these results indicated the River Nile water is unsafe and may possess a carcinogenic risk.

## 4. Conclusion

All analyzed PCBs were determined in water samples collected along the River Nile. PCBs patterns revealed that highly chlorinated PCB technical mixtures such as Aroclor 1254 were the main PCB production historical used in Egypt. Spatial trends reflected increasing in PCB levels from the upper stream to the Nile estuaries. The calculated flux of PCBs indicated that 6.8 tons of PCBs is dumped into the Mediterranean Sea each year from the River Nile. Thus, accumulation and biomagnification of PCBs residues in the aquatic ecosystems are expected. Hazard quotients and carcinogenic risk of polluted River Nile water were above the acceptable level indicating that river water is unsafe and may adverse health effects for all age groups. Based on that, stricter legislation and regulatory controls on maximum permitted levels of PCBs in industrial sectors are crucial to be implemented and emphasized by the Egyptian Environmental Affairs Agency to reduce their hazardous risks.

## Figures and Tables

**Table 1:** Mean concentrations of PCBs (*µ*g/L^−1^) in water samples from the River Nile, Egypt.

Site No.	Sampling site	Region	Mean concentration of PCB congeners (*µ*g/L^−1^)										Total PCBs
			PCB congener 28	PCB congener 52	PCB congener 44	PCB congener 70	PCB congener 101	PCB congener 153	PCB congener 118	PCB congener 105	PCB congener 138	PCB congener 180	
1	Alwasta-Beni Sweif	Greater Cairo	ND	1.188	2.222	1.089	2.165	1.462	0.821	1.704	2.482	1.370	**14.503**
2	Helwan												
3	Cairo												
4	Alaeat-Giza												
5	Giza town												
6	Alknater-Giza												
7	Qalubiya												

8	Monofeya	Nile Delta	0.161	0.917	4.391	1.867	1.211	0.951	0.188	1.412	5.322	2.351	**18.771**
9	Belbas												
10	Benha												
11	Alazezea-Menia El Kamh												
12	Menia El Kamh town												
13	Zagazig												
14	Mansoura												

15	Fraskour-Damietta	Nile Estuaries	2.708	0.158	1.866	1.411	0.924	5.189	ND	3.949	2.315	1.646	**20.166**
16	Damietta town												
17	Ras El-bar												
18	Gamasa												
19	Rosetta town												
20	Edfina-Rosetta												

Total mean of PCB congeners ± standard error			2.869 ± 0.27	2.263 ± 0.21	8.479 ± 0.47	4.367 ± 0.35	4.3 ± 0.29	7.602 ± 0.41	1.009 ± 0.11	7.065 ± 0.46	10.119 ± 0.49	5.367 ± 0.34	53.44

ND = not detectable.

Number of samples = 60 (3/each sampling site).

**Table 2:** Individual average daily doses (mg/kg^−1^/d^−1^) and hazard quotients of monitored PCBs, according to the age group.

Water sampling region	Total PCBs (ng/dm^3^)	ADD/HQ_0–6_	ADD/HQ_7–11_	ADD/HQ_Adult_
Greater Cairo	14503	284.3/**142.2**	308.9/**154.5**	284.3**/142.2**
Nile Delta	18771	367.9/**183.9**	399.9/**199.9**	367.9**/183.9**
Nile Estuaries	20166	395.3/**197.6**	429.6/**214.8**	395.3**/197.6**

Bold numbers are hazard quotients values.
